# Pharmacotherapeutic potential of pomegranate in age-related neurological disorders

**DOI:** 10.3389/fnagi.2022.955735

**Published:** 2022-09-01

**Authors:** Mohammad Javad Emami Kazemabad, Sara Asgari Toni, Neda Tizro, Parisa Alsadat Dadkhah, Hanieh Amani, Shima Akhavan Rezayat, Zahra Sheikh, Mohammad Mohammadi, Dorsa Alijanzadeh, Farnoosh Alimohammadi, Mehregan Shahrokhi, Gisou Erabi, Masoud Noroozi, Mohammad Amin Karimi, Sara Honari, Niloofar Deravi

**Affiliations:** ^1^Student Research Committee, School of Medicine, Qom University of Medical Sciences, Qom, Iran; ^2^Student Research Committee, School of Medicine, Shahid Beheshti University of Medical Sciences, Tehran, Iran; ^3^School of Medicine, Guilan University of Medical Sciences, Rasht, Iran; ^4^Student Research Committee, School of Medicine, Isfahan University of Medical Sciences, Isfahan, Iran; ^5^Student Research Committee, Faculty of Medicine, Mashhad University of Medical Sciences, Mashhad, Iran; ^6^Student Research Committee, Faculty of Medicine, Islamic Azad University of Mashhad, Mashhad, Iran; ^7^Student Research Committee, School of Medicine, Babol University of Medical Sciences, Babol, Iran; ^8^Student Research Committee, School of Medicine, Shahid Sadoughi University of Medical Sciences, Yazd, Iran; ^9^Student Research Committee, School of Dentistry, Shahid Beheshti University of Medical Sciences, Tehran, Iran; ^10^School of Medicine, Shiraz University of Medical Sciences, Shiraz, Iran; ^11^Student Research Committee, Urmia University of Medical Sciences, Urmia, Iran; ^12^Department of Biomedical Engineering, Faculty of Engineering, University of Isfahan, Isfahan, Iran; ^13^School of Medicine, Shahid Beheshti University of Medical Sciences, Tehran, Iran

**Keywords:** pomegranate, *Punica granatum* L., age-related neurological disorders, Parkinson's disease (PD), Alzheimer's disease (AD)

## Abstract

Age-related neurological disorders [AND] include neurodegenerative diseases [NDDs] such as Alzheimer's disease [AD] and Parkinson's disease [PD], which are the most prevalent types of dementia in the elderly. It also includes other illnesses such as migraine and epilepsy. ANDs are multifactorial, but aging is their major risk factor. The most frequent and vital pathological features of AND are oxidative stress, inflammation, and accumulation of misfolded proteins. As AND brain damage is a significant public health burden and its incidence is increasing, much has been done to overcome it. Pomegranate (*Punica granatum L*.) is one of the polyphenol-rich fruits that is widely mentioned in medical folklore. Pomegranate is commonly used to treat common disorders such as diarrhea, abdominal pain, wound healing, bleeding, dysentery, acidosis, microbial infections, infectious and noninfectious respiratory diseases, and neurological disorders. In the current review article, we aimed to summarize the data on the pharmacotherapeutic potentials of pomegranate in ANDs.

## Introduction

Aging is defined as the process of becoming older in which a progressive decline in human's ability occurs. The number of people aged over 60 years is growing. The number of this population measured in 2019 was 1 billion, but it is predicted that it will increase to 2.1 billion by 2050 (Chesebro et al., 2015). Aging is a key risk factor responsible for extended neurological disorders due to the vulnerability of cerebral tissue to aging consequences compare to other organs (Wyss-Coray, 2016). ANDs featured as multifactorial disorders include NDDs like PD and AD, as well as other ANDs such as epilepsy and migraine (Mattson and Magnus, 2006; Jové et al., 2014). Common pathological hallmarks of ANDs include oxidative stress, neuroinflammation, neuronal loss, and atypical protein concentration in the central nervous system (CNS) (Mattson and Magnus, 2006; Jové et al., 2014; Buendia et al., 2016). On the basis of the global burden of disease study, ANDs have been a considerable burden on the public healthcare system due to the aged population (Silberberg et al., 2015; Thakur et al., 2016). Aging is related to a peak in epilepsy incidence that can be related to brain diseases such as stroke and dementia (Beghi and Giussani, 2018). With aging, cerebral circulation alternates both structurally and functionally, which leads to higher risk of stroke incidence (Yousufuddin and Young, 2019). Common drugs used in management of neurological disorders may lead to several side effects such as constipation, dizziness, palpitation, headache, alternation in blood pressure, gastroesophageal reflux disease, sexual dysfunction, and fatigue (Cash, 1994; Bloch and Basile, 2017; Dokken and Fairley, 2020). Because of these kinds of side effects, the number of patients demanding herbal medicine is increasing not just for their lower price but also for higher cultural acceptability and better body compatibility (Wang and Ren, 2002; Pal and Shukla, 2003).

Pomegranate (*Punica granatum L*.), considered as a polyphenol-rich fruit, has been widely used in traditional medicine (Langley, 2000). It is one of the economic fruits that are native to central Asia, especially areas of Iran, from where it spreads over the world (Chandra and Babu, 2010; Verma et al., 2010). It belongs to the Punicaceae family and is one of the oldest known edible fruits (Pande and Akoh, 2016). Its name has been mentioned in the Korean and Chinese arts and the bible. Pomegranate was introduced into the culture around 5,000 years ago based on several documents from literature, archeo-botanical samples, etc. (Chandra et al., 2010). Pomegranate is known to have healing effects on prevalent illnesses, such as acidosis, stomachache, diarrhea, dysentery, bleeding, microbial infections, and various respiratory pathological conditions, and wound repair properties (Vidal et al., 2003). Previous research has shown antibacterial and anti-inflammatory advantages of pomegranate (Aviram, 2002; Aviram et al., 2004; Mori-Okamoto et al., 2004; Rosenblat et al., 2006a, 2010; Rosenblat and Aviram, 2011; Bandeira et al., 2012; Butterfield et al., 2013). As said above, pomegranate contains considerable amounts of polyphenols (flavonoids and phenolic acids), which are potent anticarcinogens and have antioxidant and anti-inflammatory effects, believed to hold back inflammation and other essential processes participating in degenerative diseases (Hirose et al., 1995; Kim et al., 2002; Aggarwal and Shishodia, 2004). The antioxidant impact of polyphenols is the most potent mechanism in charge of pomegranate protective benefits (Rosenblat and Aviram, 2011). Tannins (ellagic acid and gallic acid) found in pomegranate pericarp are strong antioxidants (Ashoori et al., 1994). They are at higher levels in pomegranate juice, made by pressing the whole fruit and pomegranate peels (Gil et al., 2000). It has been demonstrated that pomegranate juice extract decreases amyloid load and enhances cognitive behavioral deficiencies in AD mouse models (Braidy et al., 2013). Because of these kinds of extended advantages, we decided to summarize all data in the field of potential pharmacotherapeutic effects of pomegranate on ANDs.

## Method

In our study, we searched the databases of PubMed, Google Scholar, except media database, Web of Science, and ResearchGate to find all relevant articles on pharmacotherapeutic potentials of pomegranate in ANDs until December 2021. According to each database, we used particular strategies and mesh terms. Our search study is shown below:

#1 pharmacotherapeutic

#2 potentials

#3 Pomegranate

#4 *Punica granuloma L*

#5 #3 or #4

#6 age-related neurological disorders

#7 ANDs

#8 Alzheimer

#9 Parkinson

#10 dementia

#11 neurotoxicity

#12 neuroinflammation

#13 brain tumor

#14 migraine

#15 epilepsy

#16 MS

#17 multiple sclerosis

#18 oxidative stress

#19 RAS

#20 Atherosclerotic

#21 neuroprotective

#22 glioma

#23 Gliosarcoma

#24 #6 or #7 or #8 or#9 or #10 or #11 or #12 or #13 or #14 or #15 or #16 or #17 or #18 or#19 or#20 or#21 or #22 or #23

#25 #5 and # 24

We used all studies conducted on neurological disorders and pomegranates' effects on them. Then, we excluded the repeated and same ones. We choose highly associated articles by reading the subjects and abstracts of the articles. After getting the full texts of the studies, we also looked for missed articles in the reference section of the articles we found.

## Constituents

Pomegranate has white to dark purple seeds placed in a white soft, astringent membrane environed by a thick red skin or peel (Viuda-Martos et al., 2010). Pomegranate flowers contain active components such as terpenoids, flavonoids, and polyphenols (Li et al., 2008). Ursolic acid and oleanolic acid are triterpenoid parts that are also considered as main active components of pomegranate flowers (Fu et al., 2014). Peels make up about 50% of the fruit weight and are a wealthy source of bioactive components, including proanthocyanidin compounds, ellagitannins, flavonoids, and phenolics. Furthermore, it incorporates diverse minerals, especially phosphorus (P), nitrogen (N), potassium (K), sodium (Na), calcium (Ca), and magnesium (Mg), as well as polysaccharides (Viuda-Martos et al., 2010). The main compounds in pomegranate peel are punicalagin, granatin, and their derivatives, which have antibacterial activity, and *Staphylococcus aureus* is the most sensitive species based on some studies (Peršurić et al., 2020). Ellagitannins have the ability to diminish lipopolysaccharide-induced intracellular nitric oxide production. They have anti-mucositis activity as investigated in 5-fluorouracil-treated rats (Chen et al., 2022). In this regard, Hamid Cheshomi et al. demonstrated that ellagic acid (EA) could suppress proliferation and migration of gastric cancer cells. It also altered the expression and activity of MMP-2, MMP-9 (genes participating in migration), P53, BAX, APAF1, BCL2 (genes known for their role in apoptosis), iNOS, NF-κB, IL-8, and TNF-α (genes participating in inflammation). It was also reported to have a significant impact on gastric cancer without any serious side effects (Cheshomi et al., 2022).

Pomegranate also contains high concentration of polyphenols, and its antioxidant properties are much more active than any other sources of dietary polyphenols such as green tea (Sohrab et al., 2014). Hydrolyzable tannins like punicalagin, punicalin, pedunculagin, gallagic acid, ellagic acid, and anthocyanins are well-known compounds involved in the antioxidant activity of pomegranate (Sahebkar et al., 2016; Huang et al., 2017). As reported, the ellagic acid in pomegranate juice and pomegranate peel extract has a cytoprotective impact on oxidative DNA injuries and oxidative damaged living cells (Seeram et al., 2004; Akbarpour et al., 2008; Lu and Yuan, 2008). Punicalagins are familiar as the main ellagitannins in the fruit that has the ability to create ellagic acid and other polyphenols by hydrolysis (Seeram et al., 2004). High contents of phenolic acids (such as coumaric, caffeic, chlorogenic, ferulic, and gallic), non-phenolic acids, malic acid, oxalic acid, and ascorbic acid can be found in fresh pomegranate juice (Basu and Penugonda, 2009; Krueger, 2012; Gheflati et al., 2019).

**Figure 1:**
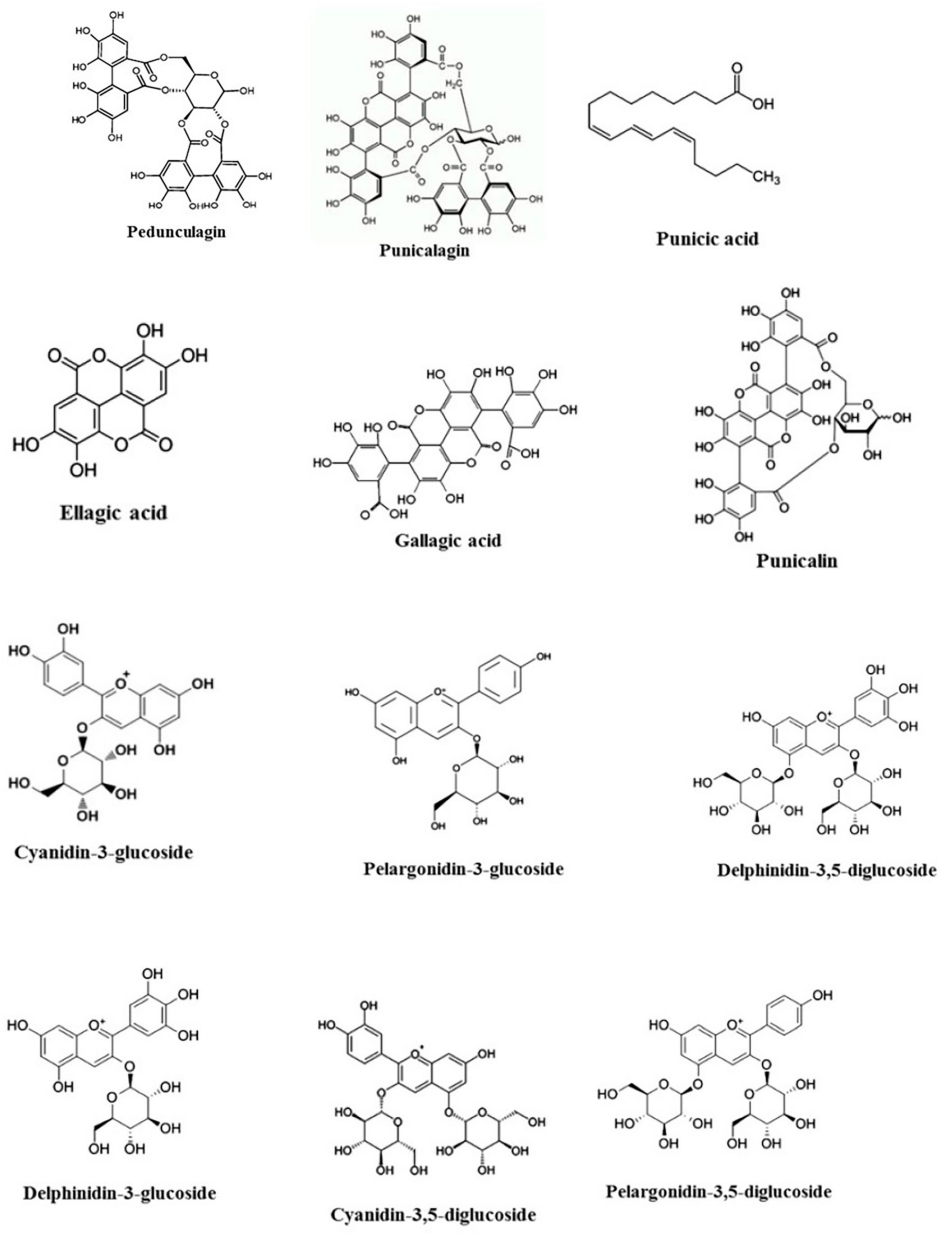
Chemical structures of main components of pomegranate.

Seeds of pomegranate have strong anti-inflammatory and antioxidant resources because of high content of hydrolyzable tannins (pedunculagin, punicalagin, esters of glucose, ellagic acid, punicalin, and gallagic acid) and anthocyanins (pelargonidin-3-glucoside, cyanidin-3-glucoside, cyanidin-3, delphinidin-3, 5-diglucoside, delphinidin-3-glucoside, and pelargonidin-3) (Afaq et al., 2005; Elfalleh et al., 2012) (Figure 1). The seeds also have multiple fatty acid properties, mainly unsaturated fatty acids such as palmitic acid, linoleic acid, oleic acid, linolenic acid, arachidic acid, palmitoleic acid, and stearic acid (Melgarejo et al., 1995; Johanningsmeier and Harris, 2011) (Figure 1). Moreover, the seed coat consists of different organic acids such as malic acid, citric acid, and ascorbic acid (Viuda-Martos et al., 2010). Additionally, pomegranate seed oil contains mostly conjugated linolenic acid. Apparently, punicic acid is a conjugated isomer of linolenic acid, which constitutes 70–76% of pomegranate seed oil (Viladomiu et al., 2013). Cerebroside, steroids, and sterols, an important constituent of the mammalian myelin sheath, represent a slight share of pomegranate seed oil (Kohno et al., 2004). Moreover, pomegranate seeds contain phospholipids that especially consist of lecithin, phosphatidylethanolamine, phosphatidylinositol, phosphatidylcholine, and lysophosphatidylethanolamine (Isamukhamedov and Akramov, 1982). The arils include water (85%), sugars (10%), mainly glucose and fructose, and pectin (1.5%). Also, arils and pomegranate juice are wealthy sources of bioactive constituents such as flavonoids, phenolics, and especially anthocyanins (Gil et al., 2000; Viladomiu et al., 2013).

Pomegranate leaves comprise some specific tannins such as those containing glycosides of apigenin, which is a flavone with anxiolytic and progestinic properties (Paladini et al., 1999; Zand et al., 2000). Furthermore, pomegranate leaves is a strong source of K, N, Fe, and Ca, but the quantity of these elements changes with season and the phase and maturation of the plant (Lansky and Newman, 2007). For instance, K amount is high in young pomegranate leaves, while Fe and Ca levels are reported to be highest in old leaves. Medium-age plants contain high N amount. However, the content of N is decreased at the time of flowering and setting of the fruit. N content is similarly reported to be reduced with maturation of the fruit (Munde et al., 1980, 1981).

## Alzheimer's disease and memory

There is a close relationship between dementia and the process of aging. The incidence and prevalence rates of dementia are higher in the elderly. The incidence rate doubles every 5 years in individuals between 65 to 95 (Kravitz et al., 2012). In natural aging, cellular waste accumulates in tissues, including the brain's, and gray matter volume gradually decreases. Brain microinfarcts are also present in an aging brain (Rosa et al., 2020). Biological changes during the process of aging lead to neuron dysfunction and are responsible for the higher incidence of this disease in the elderly. Furthermore, older people are more likely to be physically inactive, making the progression of the disease faster (Bich et al., 2019).

Alzheimer's disease (AD) is an age-related cognitive disorder with the hallmark of neurofibrillary tangle accumulation and subsequent atrophy of the hippocampus, amygdala, and cortex. Clinical symptoms are associated with the degree of tau deposition. Amyloid-β deposition is also involved in the pathophysiology of the disease (Sengoku, 2020). Pomegranate extract has a role in modification of Alzheimer's disease in several mechanisms. A previous publication by Braidy et al. on pomegranate and synaptic activity in Alzheimer mouse models confirmed enhanced synaptic plasticity and anti-inflammatory effects by reduction of amyloid-beta and relevant precursor protein cleavage in mouse models (Braidy et al., 2016). In an *in silico* study by Yuan et al., pomegranate extract compounds and their ability to penetrate the blood-brain barrier showed that only ellagitannin gut microbiota-derived compounds, urolithins (6 hdibenzo[b,d]pyran-6-one derivatives) met the criteria and prevented amyloid-beta fibrillation and neural death in AD (Yuan et al., 2016). Another publication on pomegranate extract effects on aged AD mouse models from Ahmed et al. (2014a) confirmed the modified activity of the γ-secretase enzyme and subsequent alternation of amyloid-beta 42/amyloid-beta 40 ratios and its inhibitory activity on amyloid-beta production without significant cognitive promotion. Pomegranate can also reduce the predisposition to AD *via* antioxidative procedures. Choi et al. experimented with the ethanol extract of 4% pomegranate on induced amyloid-beta PC12 cell lines (rat pheochromocytoma cells). The results showed antioxidative and anti-neurotoxicity effects due to the anti-amyloid beta activity of the 2,4-di-tert-butylphenol compound in the extract (Choi et al., 2011). A clinical trial compared the effects of Pomegranate juice with those of placebo juice on 28 elderly individuals with memory complaints. The investigators found increased memory performance in the older people who consumed pomegranate juice. Furthermore, the fMRI results of the pomegranate group showed increased cerebral blood flow during the tasks compared with the placebo group. Altogether, the researchers stated that the antioxidant properties of polyphenols in pomegranate juice might augment memory function by increasing functional brain activity (Bookheimer et al., 2013). According to an *in vitro* study by Khokar et al., pomegranate peel extract has antioxidant agents and inhibits the acetylcholinesterase (AChE) enzyme. Therefore, they suggested pomegranate as a natural alternative antioxidant source with anti-AD benefits (Khokar et al., 2021).

In rats with AD induced by aluminum chloride (AlCl_3_), pomegranate juice reverted some aluminum effects on memory and learning abilities. This group had fewer errors of entry and better results in the water maze test than the group that was not treated with pomegranate. The mice's histopathological assay suggested that the neuroprotective benefits of pomegranate might be related to the decrease in the number of glial cells in the hippocampal region (Abdulmalek et al., 2015). Another study on AD mouse models found that the group fed with punicalagin and ellagic acid had better results in Barnes maze and T-maze. Furthermore, according to *in vitro* observations, these compounds showed anti-inflammatory effects by decreasing microgliosis and inhibiting the nuclear factor of activated T-cell (NFAT) activity and TNF-α (tumor necrosis factor-alpha) secretin (Rojanathammanee et al., 2013). Mahsan Akbarian et al. examined the impact of pomegranate seed hydro-ethanolic extract on scopolamine-induced amnestic rats. The extract reduced learning problems in the rats, possibly by balancing acetylcholinesterase function, oxidative stress, and expression of inflammatory cytokines such as TNF-α, and IL-1β (Akbarian et al., 2022).

**Figure 2:**
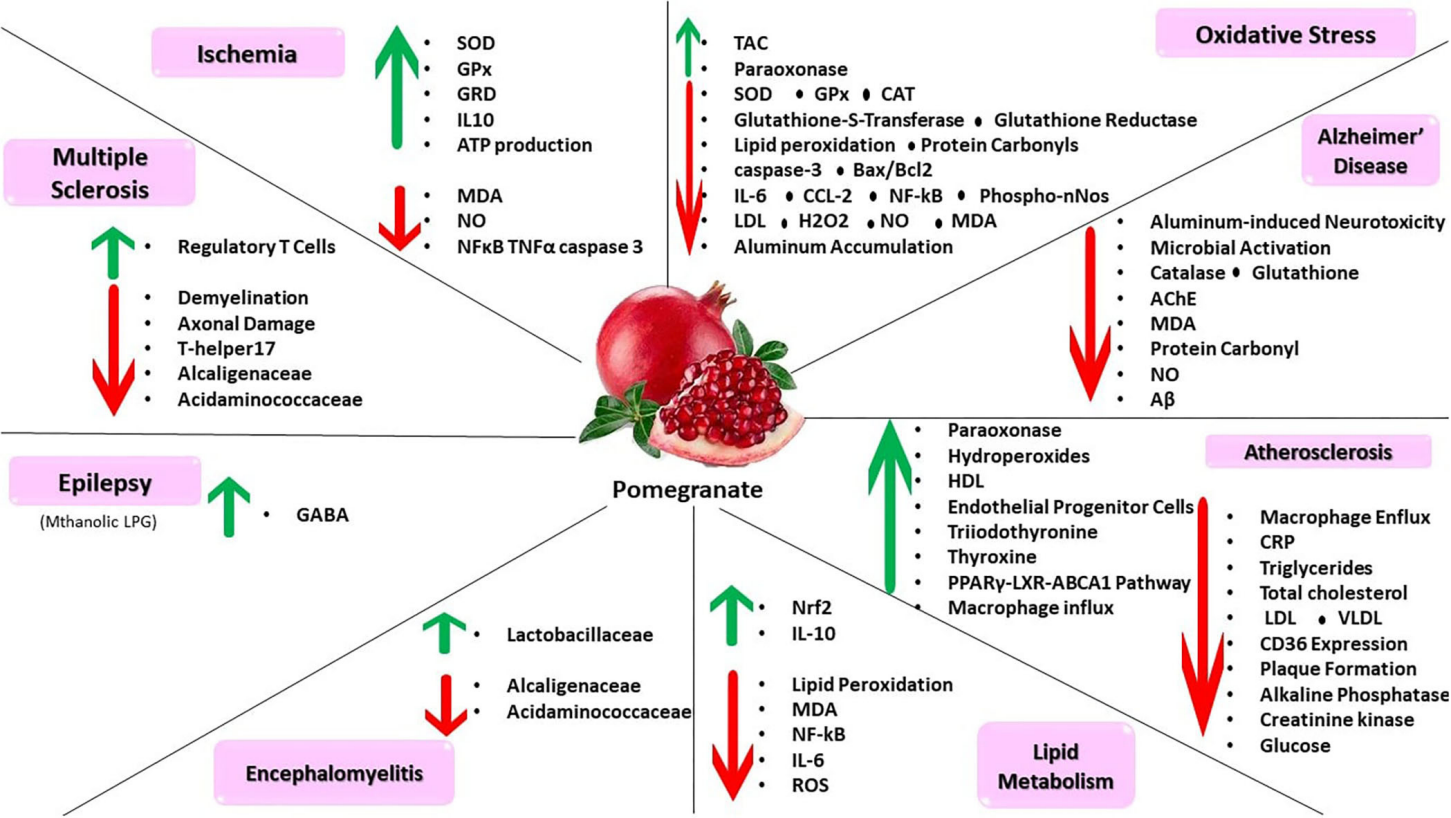
Pomegranate can inhibit oxidative stress, atherosclerosis, lipid metabolism, and encephalitis. This compound has protective agents for age-related neurological disorders such as Alzheimer's disease, epilepsy, multiple sclerosis, and ischemia.

Pomegranate and its nanoparticle formulation are also shown to have potential benefits in increasing brain-to-body weight ratio in AD mouse models. Mice treated with pomegranate extract had a better cognitive function, and a biomarker assay showed enhanced catalase and total antioxidant capacity. According to a histopathological examination of mice brains, pomegranate nano-formulation may also reduce pathological features (Almuhayawi et al., 2020). Feeding mice with pomegranate caused a decrease in lipid peroxidation (LPO). It augmented the activities of several antioxidant enzymes, e.g., catalase, superoxide dismutase (SOD), glutathione peroxidase (GPX), and glutathione-S-transferase (GST), which may indicate reduction in oxidative stress levels of cells. Therefore, the investigator suggested that patients with AD might benefit from this fruit (Subash et al., 2014). However, in their *in silico* study, Almuhayawi et al. stated that compounds found in pomegranate juice and their metabolite might inhibit catalase, SOD, GST, glutathione reductase (GRD), and GPX4. Inhibition of these enzymes may be responsible for increasing cellular oxidative stress levels, thereby causing adverse events in patients. The authors concluded that phytochemicals in pomegranate juice might have pro-oxidant effects along with their antioxidant potency, and they cautioned about using pomegranate juice in patients with NDD who are prone to oxidative stress (Mazumder et al., 2019). Ramasamy et al. assessed the *in vivo* neuroprotective effect of the ethanol, and chloroform extracts of the *Punica granatum L.var* leaf, by using the model organism amyloid b protein of *Drosophila melanogaster*. The leaf extract significantly reduced the adverse morphological alternations of amyloid β protein in *Drosophila* by improving motor skills, rescuing neurodegeneration, and increasing the lifespan in amyloid β protein-expressing Drosophila. Therefore, this study showed that the use of ethanol and chloroform extracts remarkably rescues, protects, and restores the impaired movement activity of *Drosophila melanogaster* in AD (Figure 2).

## Atherosclerosis

Atherosclerosis is an age-related disorder characterized by atherosclerotic plaque formation with telomere dysfunction, DNA damage, and increased apoptosis with reduced cell proliferation (Wang and Bennett, 2012). Hasan Noor Ibrahem designed a study to assess the potential effect of pomegranate juice and an aqueous extract on cholesterol-induced atherosclerosis and H_2_O_2_-induced oxidative stress in rat models. The total range of cholesterol and Alklsaredat triple significantly decreased in juice and extract-treated oxidative and atherosclerotic environment (Hasan, 2019). Atherosclerotic plaques are formed by a fibrous cap full of vascular smooth muscle cells (VSMCs) and collagens, covering a necrotic core including lipids, foam cells, and debris. Plaque development consists of serial events starting from increase in adhesion molecules, including vascular adhesion molecule 1 and intracellular cell adhesion molecule 1, on the surface of epithelial cells. Additionally, improvement in vascular permeability causes infiltration of lipid and inflammatory cells into the sub-endothelial area. Macrophages are differentiated forms of monocytes that absorb lipids to develop foam cells. These cells secrete pro-inflammatory cytokines to form an inflammatory setting. In the early stages, VSMCs increase and immigrate to produce fibrous caps, but in late stages, aged VSMCs secrete various cytokines, deteriorating the pre-existing inflammation. Therefore, aging is considered a crucial risk factor for atherosclerosis (Wang and Bennett, 2012).

The effect of antioxidant compounds of pomegranate juice on antioxidant and enzyme activities was investigated by El Hussieny et al. in atherosclerosis development on white rabbit models. Paraoxonase1 enzyme, total antioxidant capacity, and HDL-c were significantly increased in the serum of pomegranate juice-treated rabbits. In contrast, triglycerides, C-reactive protein, total cholesterol, VLDL-c, and LDL were significantly decreased in this group (Al-hadidy et al., 2014). Al-Hadidy et al. showed that pomegranate peel extracts abundant with polyphenol products have an anti-atherosclerotic effect by inhibition of hyperlipidemia and oxidative stress, and CD36 expression modification (El Hussieny et al., 2018). Ahmadi et al. explored the antioxidant effect of pomegranate peel extract on an atherosclerotic rabbit model, and found increased and modulated number of endothelial progenitor cells and inhibition of plaque formation in the aorta (Ahmadi et al., 2012). The majority of research has declared that pomegranate has shown a great potential to stop hyperlipidemia by affecting serum lipid profiles and alternation in gut microbiota. Moreover, it can prevent and revere atherosclerosis by changing macrophage influx and efflux rate of Ox-LDL, increasing paraoxonase activity, and reducing cholesterol esterification. Also, it can have positive effects on cardiovascular diseases, thyroid dysfunction, glucose hemostasis, and fatty liver. Atherosclerosis is one of the leading causes of cerebrovascular disease, ischemic stroke, and heart attack worldwide and has increasing prevalence (Herrington et al., 2016). Hypercholesterolemia and hyperlipidemia, mainly promoting levels of oxidized LDL and plasma LDL cholesterol, are crucial initiation factors in the development of atherosclerotic lesions (Weber and Noels, 2011). One of the animal atherogenesis models, known as the atherosclerotic apolipoprotein E–deficient mouse, has shown severe hypercholesterolemia development on a diet with low cholesterol that contributed to increase in extensive atherosclerosis and oxidative stress (Jawien et al., 2004). Magdalena et al. showed the protective effect of conjugated linolenic acid in pomegranate seed oil on atherosclerosis in ApoE/LDLR-/-mouse models by reduction of total cholesterol (Franczyk-Zarów et al., 2013). Parmar et al. examined the effect of *Punica granatum* peel extract on diet-induced thyroid dysfunction and atherosclerosis in rats. Amazingly, after administration of these fruit extract peels to atherosclerosis rat models, the levels of serum lipids including total cholesterol, triacylglycerol, LDL and VLDL cholesterol, atherogenic index, alkaline phosphatase, and creatinine kinase-MB were decreased, with a parallel increase in HDL, thyroid hormones, triiodothyronine and thyroxine, and serum level of insulin and a concurrent reduction in serum glucose. Also, histopathologic alterations like fatty liver, cardiac muscle hypertrophy, and mild renal tubular damage were reversed, which suggests that the anti-oxidative, anti-inflammatory, and anti-atherogenic potentials of the peels are attributed to high levels of phenolic compounds, flavonoids, and ascorbic acid (Parmar and Kar, 2008). Furthermore, this study reported a protective effect of the fruit extract peels on cardiovascular diseases and thyroid dysfunctions. Paradisiaca was not as effective as *C. sinensis* and *P. granatum* peels (Parmar and Kar, 2007).

Kaplan et al. studied the useful impacts of a tannin fraction derived from pomegranate juice (PJ) in apolipoprotein E-deficient (E0) mice that had severe atherosclerosis. This study showed that administration of PJ in E0 mice interestingly lessened the oxidized (Ox)-LDL mouse peritoneal macrophage (MPM) uptake by 31%, reduced MPM cholesterol esterification, increased macrophage cholesterol efflux by 39%, and decreased lesion size by 17% in comparison with placebo-treated mice (Kaplan et al., 2001). PJ plays its anti-atherogenic effect by increasing serum paraoxonase activity by hydrolyzing peroxides and cholesteryl linoleate hydroperoxides (Aviram et al., 2000b). Also, it decreases the macrophage influx rate of Ox-LDL *via* scavenger receptors with a parallel increase in macrophage efflux rate (Berliner et al., 1995; Aviram, 1996). Besides, it is seen that cholesteryl ester droplets have been reduced after PJ consumption (Maor and Aviram, 1994; Ross, 1999). Rom et al. evaluated the protective impacts of polyphenol-rich PJ on acrolein in E0 mice. Acrolein is a pro-atherogenic factor derived from different types of food (Abraham et al., 2011), and it is known to elevate the possibility of cardiovascular diseases and atherosclerosis formation (DeJarnett et al., 2014). After consumption of PJ by mice that received acrolein, they exhibited a notable reduction in triglycerides, aortic and serum cholesterol, and lipid peroxides. Moreover, it dissolved cholesterol and triglyceride accumulation in peritoneal macrophages. Additionally, it normalized the alternation in gut microbiota, which had been aroused by acrolein. At the phylum level the number of firmicutes had improved and the number of Bacteroidetes had reduced (He et al., 2008). At the family level, the amount of Ruminococcaceae and Lachnospiraceae had developed. The coprococcus genus from the Lachnospiraceae family is believed to increase serum, aortic, and macrophage lipid ranges and peroxidation (Rom et al., 2017).

On the other hand, Radjabian et al. investigated the impacts of pomegranate juice and its seed oil on hypercholesterolemic rabbits fed with high-cholesterol diet including 1% cholesterol. This 2-month study revealed that although pomegranate juice and its seed oil (either at 1 or 2% dose) decreased atherosclerotic plaque development in hypercholesterolemic rabbits' aortas, they did not affect serum lipid levels significantly (Radjabian et al., 2008a,b). Aviram et al. (Aviram et al., 2000a) showed that pomegranate juice administration reduces native LDL and oxidized LDL uptake by peritoneal macrophage cells by 20% and attenuates the size of the atherosclerotic lesion by 44% in E0 mice. Additionally, Kaplan et al. (2001) reported that a tannin fraction isolated from pomegranate juice decreases atherosclerosis. Aviram et al., in another investigation (Aviram et al., 2008), evaluated the antiatherogenic features of various parts of pomegranate extracts in J774A.1 macrophage cells and E0 mice. In atherosclerotic apolipoprotein E–deficient mice, pomegranate flower extract demonstrated the most significant impact, as it considerably attenuated the area of atherosclerotic lesions by 70% and decreased the glucose and serum lipid levels by 18–25%. In J774A.1 macrophages, various tested pomegranate extracts were related to the same antiatherogenic effects as some pomegranate phenolics (Allahverdian et al., 2014). Elbandy et al. showed that a diet comprising 10% pomegranate seed residue, one with 5% pomegranate seed oils, and one with a 15% combination of both of them decreases the plasma levels of LDL cholesterol, total cholesterol, and triglyceride (Elbandy, 2012).

Pomegranate ellagic acid, punicalagin, and pomegranate peel polyphenols reduce the total cholesterol levels of human embryonic liver cell line L02 by suppressing the synthesis of cholesterol (Lv et al., 2016). Also, polyphenols prevent early atherogenesis in RAW264.7 macrophage cells by upregulating the LXRα/PPARγ-ABCA1 pathway (Zhao et al., 2016). Oxidative modification of LDL is another initiating factor in atherogenesis. Oxidizing LDL by upregulating adhesion molecules in endothelial cells and inducing the expression of chemotactic agents in endothelial cells contribute to the development of inflammatory processes and atherosclerotic lesion formation (Monguchi et al., 2013). Bagri et al. (2009), in their *in vitro* study, showed that pomegranate wine had a more antioxidant effect than Cabernet Sauvignon red wine on preventing LDL oxidation, which may be related to high phenol levels.

## Prion diseases

Human prion disease manifestation is more seen in the elderly. The median age of death from the disease in the United States is 67 years, and patients younger than 30 years are extremely rare (Maddox et al., 2020). The incidence of the disease dramatically increases with age and peaks at 70 to 79 years, suggesting that older people are more involved than the young. Because of low survival rates and unfavorable prognosis of the disease, prion should be regarded as one of the life-threatening diseases in the elderly (Sun et al., 2020). Studies suggest that conversion of normal prion protein (PrP^C^) to pathogenic prion scrapie protein (PrP^C^) is the basis of disease pathogenesis. During the process of aging, catabolism of PrP^Sc^ might be impaired (Goh et al., 2007; Chesebro et al., 2015), and proteins gradually accumulate in tissues, including the brain's. Protein accumulation provides the basis for oxidative stress, inflammation, and disease development (Trigo et al., 2019). According to the study conducted by Mizrahi et al., administration of nano-PSO, a nanodroplet compound of pomegranate seed oil (PSO), had potential benefits in delaying disease manifestation and exacerbation in TgMHu2ME199K mice. It showed neuroprotective features by inhibiting neuron death and increasing neurogenesis in the hippocampus. Administration of the nanodroplet decreased lipid oxidation but did not change mutant PrP expression or accumulation in the brain. Therefore, the investigators suggested nano-PSO as a safe and effective compound to be used for individuals with NDD (Mizrahi et al., 2014).

## Oxidative stress

Several researchers have attempted to explore pomegranate compound impacts on oxidative stress and brain cell toxicity. Annaç et al. (2022) observed the outcomes of exposure to pomegranate juice in lead acetate-induced neurotoxicity. Pomegranate juice alleviates the subsequent adverse effects of hippocampal degeneration in male rats. Moreover, malondialdehyde levels and glutathione S-transferase activity were significantly decreased. In another study, Foroutanfar et al. explored the properties of punicalagin (the primary polyphenol found abundant in pomegranate) in industrial acrylamide induced-neuro- and hepatotoxicity in rats. The results show ameliorated movement disabilities, reduced apoptotic characteristics *via* decreased caspase 3 and Bax/Bcl2 ratio, attenuated oxidative patterns in the brain and liver through lower glutathione and malondialdehyde levels, and increased myelin basic protein with following lesser acrylamide toxicity (Foroutanfar et al., 2020). Ginsberg et al. determined the impact of pomegranate juice on inflammatory and apoptotic pathways in rats. The authors found that pomegranate polyphenols modified the maternal brain's inflammatory environment by reducing pro-inflammatory cytokines (IL_6 and CCL_2), apoptosis, and oxidative stress through reduced caspase 3, NFkB p 65, and phospho-nNos protein in fetal brains (Ginsberg et al., 2017). Many countries have faced growth in their elderly population. Since the process of aging process of considerable importance for atherosclerosis and diseases like that (Edo and Andrés, 2005), it is highly recommended to prevent such diseases by consuming natural compounds like pomegranate.

Lack of balance between the generation and aggregation of oxygen reactive species (ROS) in tissues and cells, as well as detoxification capability of these reactive products in a biological system, causes oxidative stress (Pizzino et al., 2017). A considerable amount of data has been collected that show the correlation between the process of human aging and the oxidant status of organisms (Junqueira et al., 2004). Seeram et al. (2004) revealed that the juice of *Punica granatum* has the highest anti-oxidative and anti-proliferative assets than total pomegranate tannin, punicalagin, and ellagic acid, illustrating a synergic biochemical property for pomegranate polyphenol compounds. In a study by Cervantes-Anaya et al. (2022), the aqueous extract of *P. granatum* and its purified ingredients (punicalagin and ellagic acid) mitigated oxidative stress and boosted brain cell functions in an ovariectomized rat model. Aviram et al. compared the inhibitory factors of aqueous solutions of crushed seeds and the outer and inner peels of the pomegranate fruit based on an equal concentration of polyphenol by analyzing the anti-oxidant features of pomegranate components other than the juice. The outcome was that the aqueous extracts of outer and inner peels were more potent anti-oxidants than the juice, which suggests that more powerful anti-oxidant polyphenols are placed in the outer and inner peels. Polyphenolic flavonoids affect cellular oxygenases and create conformational shifts in plasma membrane components, like lipoprotein cellular receptors. After consumption of pomegranate juice, the absorption of native LDL and oxidized LDL *via* macrophages of the peritoneum from E° mice was analyzed. Cellular binding, cell association, and cellular degradation of both native LDL and oxidized LDL were lower in cells from mice nourished with pomegranate juice than in cells from the control group. As atherosclerotic E° mice consumed pomegranate juice, cell oxidative stress and uptake of oxidized LDL were decreased (Aviram et al., 2000a). It was stated in another article that pomegranate juice and seed oil extract can have a positive effect on decrease in the formation of aortic atherosclerosis plaques (Radjabian et al., 2008a,b). In a study by Sun et al. (2017), ellagic acid (a polyphenolic compound from pomegranate peels) showed more anti-oxidative effects on intestinal free radicals in *in vitro* models, and multiple mixtures of peels' polyphenolic compounds had more significant anti-stress effects than single ones. It was reported that pomegranate could improve antioxidant factors like total serum antioxidant capacity, superoxide dismutase, and malondialdehyde (Lorzadeh et al., 2022).

Atherosclerosis is categorized as a disease of aging. It is said that aging is one of the independent risk factors for atherosclerosis development (Wang and Bennett, 2012). Stress-induced myocardial ischemia in patients with coronary artery diseases is improved when pomegranate juice is consumed daily, as was shown recently (Sumner et al., 2005). In a comparison conducted by Rosenblat et al., the concentration of entire polyphenols in a pomegranate by-product was higher than in pomegranate juice. The pomegranate by-product was consumed by E° mice for 3 months. The outcome was considerable decrease in the size of the lesion of their atherosclerotic in comparison to the placebo group's lesion size (Rosenblat et al., 2006b). Al-Jarallah et al. analyzed the effects of inflammation and oxidative stress on advancement of occlusive atherosclerosis of coronary artery, spontaneous aortic sinus, and myocardial infarction in scavenger receptor, class-B, type-I (SR-BI)/apoE dKO mice. It was revealed that only after 3 weeks of age in SR-BI/apoE double knockout mice atherosclerosis in the coronary arteries and aortic sinus, enlargement of the heart, and fibrosis of the heart start developing. When the treatment process of these mice started with pomegranate fruit extract at 3 weeks of age, the result was reduced coronary artery atherosclerosis and decreased cellular oxidative stress in artery walls (Al-Jarallah et al., 2013). It was stated in another article that consumption of pomegranate can protect cardiovascular health by increasing nitric oxide that assists in the function of lining cells of the arterial wall (Ignarro et al., 1999). Al is one of the most well-studied neurotoxins that affect the nervous system, including different brain areas (Nehru and Bhalla, 2006). Al crosses the blood-brain barrier and plays an important role in neurofibrillary tangle formation in AD (Sharma et al., 2009). Abdel Moneim, in his research, investigated the effect of pomegranate peels on aluminum-induced oxidative stress and followed histopathological changes in the brain of female rats. The study showed that it is an indicator of carcinogenicity in the AlCl3 treatment group and represents an elevation of tissue tumor markers, including angiogenin and tumor necrosis factor α, and inflammation by increasing prostaglandin F2a and prostaglandin E2 levels. Pomegranate peel methanol extract protects the brain by reducing aluminum accumulation and stimulating antioxidant activity and the anti-apoptotic protein, i.e., Bcl2. These findings indicate that pomegranate extract may suppress histopathological changes and aluminum-induced oxidative stress in the brain of female rats, which may be associated with anti-apoptotic and antioxidant agent activity (Abdel Moneim, 2012).

In another project, he conducted an investigation on the antioxidant properties of pomegranate peel extract on the brain of rats. The methanol extract of *P. granatum* skin was assayed for its antioxidant activity in the brain of adult rats. Antioxidant activity was determined by measuring reduced glutathione peroxidase, glutathione transferase, glutathione reductase, superoxide dismutase, catalase, and glutathione. In addition, lipid peroxide (MDA), nitric oxide (NO), and hydrogen peroxide (H2O2) were also measured with brain homogenates. Treatment with pomegranate skin reduced the oxidants H2O2, NO, and MDA, and significantly increased most antioxidant parameters. It may be concluded that pomegranate methanol peel extract may help prevent neurodegenerative disorders caused by oxidative stress (Moneim, 2012). It has been suggested that oxidative stress is a molecular disorder caused by free radical buildup and a direct cause of aging (Harman, 1992). This condition may generate oxidative damage in various biomolecules as well as changes in redox homeostasis, leading to emergence of age-related illnesses (Saretzki and Von Zglinicki, 2002; Forman, 2016; León Regal et al., 2018; Liguori et al., 2018). Pomegranate peel is classified as an agricultural waste; however, it is a rich source of chemicals that benefit human health. The peel contributes around 60% of the weight of pomegranate (Lansky and Newman, 2007). The pomegranate peel has higher total phenolic material and antioxidant function than pomegranate pulp (Li et al., 2006). The peel has stronger antioxidant effects than the flower, leaf, and seed (Elfalleh et al., 2012). Polyphenols of the peel have the potential to remove radicals, i.e., they have antioxidant properties. Production of free radicals is critical in the development of a variety of clinical conditions such as atherosclerosis (Steinberg, 1988), brain dysfunctions (Gordon, 1996), and cancer (Halliwell and Gutteridge, 2015). They can also suppress cell growth and promote apoptosis in human prostate cancer cells (Lansky et al., 2005). Green pomegranate peel extract showed anticancer properties in human lung adenocarcinoma and human colorectal carcinoma cells *in vitro*. Immature peel is an abundant source of polyphenols, particularly gallotannins like granatin B. Granatin B has antiproliferative and apoptotic activities against tumoral cells (Russo et al., 2021). Since punicalagin and granatin B arrest cells in the S-phase, they seem to be a good therapeutic agent against colorectal cancer (Chen et al., 2022). The proliferation of glioma and lung cancer cells *in vitro* was also inhibited by granatin B (Jin et al., 2016; Toda et al., 2020).

Pomegranate contains several flavonoids, specifically punicalagin, and it has been shown to have an inhibiting impact on the influenza virus. Therefore, it can be concluded that pomegranate extract may suppress the activity of viruses transmitted to the body (Howell and D'Souza, 2013). Pomegranate seeds, juice, and leaves have antioxidant properties. The fruit rind, bloom, and leaves are analgesic and anti-inflammatory (Rahmani et al., 2017). In an animal study on ovaries of rabbit, Bakeer et al. showed that pomegranate peel supplementation increased total anthocyanin content (TAC) and superoxide dismutase (SOD). It also reduced malondialdehyde (MDA) significantly (Bakeer et al., 2022). Morvaridzadeh et al. (2020) reported about malondialdehyde (MDA), plasma glutathione peroxidase (GSHPxis), total anthocyanin content (TAC), paraoxonase, and thiobarbituric acid reactive substances (TBARSs). This study showed that eating pomegranate slightly reduced MDA levels but had no considerable effect on glutathione peroxidase levels. GSHPxi is one of the antioxidant markers in serum and plays a crucial role in reactive oxygen species (ROS) absorption (Raes et al., 1987). The polyphenols in pomegranate can reduce ROS levels, so less glutathione production is needed in the body for antioxidant protection by eating this fruit (Faria et al., 2007). This study also demonstrated that pomegranate caused a slight elevation in TAC levels and caused a slight increase in paraoxonase levels (Morvaridzadeh et al., 2020). However, according to a systematic review and meta-analysis, the effect of pomegranate on TAC level and GSH-Pxis was not found to be significant (Morvaridzadeh et al., 2020). The elevated paraoxonase after ingestion of pomegranate confirms the antioxidant properties of pomegranate polyphenols, including potent ROS scavengers such as ellagic acid, anthocyanins, and tannins (Gil et al., 2000; Rock et al., 2008). The effect of pomegranate intake on urinary TBARSs was also assessed in former studies (Gouda et al., 2015).

## Lipid metabolism mechanism regulated by pomegranate

An investigation conducted on Wistar rats demonstrated that aqueous pomegranate peel extract consumption decreased lipid peroxidation in renal, hepatic, and cardiac tissues (Parmar and Kar, 2008). Feeding mice with a high-fat diet can cause demyelination of granular and Purkinje cells in the brain cortex. Mice fed with pomegranate juice showed improved function. Pomegranate supplementation decreased caspase immunoreaction and ameliorated the cortex damage induced by hyperlipidemia in mice (El-Sayyad et al., 2020). Consumption of pomegranate juice reduced the plasma levels of malondialdehyde, one of the most critical indicators of intracellular lipid peroxidation in healthy individuals (Matthaiou et al., 2014); more considerably, plasma levels of malondialdehyde in patients with type 2 diabetes mellitus also remarkably declined after treatment with pomegranate juice (Sohrab et al., 2015). Moreover, the anti-lipid peroxidation features of pomegranate are significantly associated with cellular antioxidant capacity improvement (Bagri et al., 2009; Matthaiou et al., 2014; Sohrab et al., 2015; Al-Gubory et al., 2016). Cells can coordinate the expression of genes that encode phase II detoxifying enzymes by activating the transcription factor Nrf2 in response to oxidative stress (Bryan et al., 2013). Nrf2, by binding to promoters of response elements, induces transcription of phase II detoxifying enzymes (Copple et al., 2008). Pomegranate extract, which contains 40% punicalagin, can activate Nrf2 and its downstream antioxidant enzymes (Sun et al., 2016; Yan et al., 2016). The up-regulation of Nrf-2 mediated by punicalagin decreases oxidative stress and improves hyperlipidemia. Therefore, punicalagin can be helpful in treating patients with impaired hepatic lipid metabolism (Cao et al., 2020). Pomegranate extract was shown to have antifibrotic effects on the liver. The extract's hepatoprotective properties were associated with modulation of the NF-κB, Nrf2/HO-1, and TGF-β/Smad3 pathways (Gowifel et al., 2020). Tumor necrosis factor inhibits the activity of lipid metabolism-related enzymes as well as increases lipolysis and lipogenesis (Chen et al., 2009). In an *in vivo* study performed by Mandal et al. (2017), pomegranate extract inhibited NF-κB translocation from the cytosol to the nucleus.

Harzallah et al. (2016) revealed that pomegranate seed oil, flower, and peel are able to reduce plasma interleukin-6 and tumor necrosis factor levels in high-fat-diet-induced obese mice, and that the flower of pomegranate increases the level of interleukin-10, which is an anti-inflammatory cytokine. Mitochondria are important cellular inflammation activation regulators and are main parts of reactive oxygen species production (Yan et al., 2013; Candas and Li, 2014). A study revealed that punicalagin and ellagic acid directly decrease the levels of reactive oxygen species manufactured by mitochondria (Zou et al., 2014). Ellagic acid exerts an antioxidative effect by ameliorating mitochondrial dysfunction in oxidative stress mediated by gentamicin (Sepand et al., 2016) and cytotoxicity mediated by arsenic trioxide in human neuroblastoma cells (Firdaus et al., 2018). Cao et al. (2015) showed that a pomegranate extract with 40% punicalagin significantly prevented cardiac mitochondrial disability mediated by a high-fat diet. In line with these findings, mitochondria are one of the targets of pomegranate extract in lipid metabolism regulation in metabolic disorders. However, specific regulatory sites of pomegranate extract on mitochondria are unknown, and further investigations on this subject in the future can give us a better insight.

## Encephalomyelitis

Experimental Autoimmune Encephalomyelitis is multiple sclerosis in animal models and an immune-mediated demyelinating condition. Age-related oxidative stress upregulation is an essential etiology to develop the disease (Seo et al., 2015). Lu et al. examined the therapeutic effects of oral pomegranate peel extract (100 mg/kg/day) on an experimental autoimmune encephalomyelitis model. The results showed a noteworthy delay in the progression of fecal microbiota in a transplantation-treated experimental autoimmune encephalomyelitis mouse model. Moreover, the gut microbiota had increased Lactobacillaceae and decreased Alcaligenaceae and Acidaminococcaceae, which alleviated symptoms resulting from the lower inflammatory state of the central nervous system and myelin loss (Lu et al., 2020).

## Epilepsy

Epilepsy is a prolonged predisposition to make epileptic seizures with neurologic side effects. There has been a strong Q25 relationship between epilepsy and age-related brain disorders like dementia and stroke (Kotloski et al., 2019). Viswanatha et al. (2016) investigated the extract of P. granatum leaves, including methanolic leaf extract of P. granatum (MLPG), petroleum ether leaf extract of P. granatum (PLPG), and aqueous leaf extract of P. granatum LPG (ALPG) in epilepsy models. Only MLPG had a significant dose-dependent anticonvulsive activity; also, the author found a correlation between MLPG anticonvulsive effects and raised brain GABA levels.

## Multiple sclerosis

Multiple sclerosis (MS) is a demyelinating autoimmune disease of the CNS in young adults. Aging-related degeneration, inflammation, and vascular mechanisms are known pathologies involved in disease progression (Sanai et al., 2016). Recent studies showed that the average age of MS is developing (Marrie et al., 2010). Also, aging is considered to be one of the main Q25 factors determining the course of MS. It is more possible for patients with MS aged over 65 to experience a progressive course (Minden et al., 2004). Aging is accompanied by systemic low-grade inflammation (Candore et al., 2010). Additionally, immune response changes or becomes unbalanced with aging (Kleinewietfeld and Hafler, 2014). In a pilot study, Petrou et al. (2021) investigated the effect of Granagard (a neuroprotective supplement in the animal model made from pomegranate seed oil) and punicic acid (the major antioxidant in pomegranate seed oil) on multiple sclerosis cognitive function. No adverse effects were seen during the study, and all the patients revealed positive activities of daily living. It also showed a cognitive improvement that lasted for at least 3 months. Lu et al. evidenced that everyday use of oral pomegranate peel extract (PPE) can notably decrease the clinical score, demyelination, and axonal damage in MOG35-55-induced experimental autoimmune encephalomyelitis mice. Moreover, the PPE treatment restrained the activation of dendritic cells, microglia, and macrophages, suppressed the production of T-helper17 cells, and increased the amount of regulatory T cells (Tregs); it is suggested that CD4+ T cells producing IL-17 and IFN-γ are the leading cause of MS, and that Tregs can restrain immune responses (Yadav et al., 2015). In this study, the result group exhibited an increase in the amount of gut microbiota, especially in the number of Lactobacillaceae, Prevotellaceae, Lachnospiraceae, and Ruminococcaeae. Earlier studies have evidenced that Lactobacillaceae has the ability to lessen the severity of EAE (Wilck et al., 2017). Additionally, the amount of Alcaligenaceae and Acidaminococcaceae showed a decrease. Earlier studies have introduced various numbers of Alcaligenaceae as opportunistic microbiota were notably increased in children with autism (Williams et al., 2012). Altogether, the results manifested that PPE can relieve symptoms of MS by alternation of gut microbiota, which restrains the production of pro-inflammatory cytokines and develop the expression of anti-inflammatory cytokines (Lu et al., 2020).

## Inflammation

### Neuronal cells

Velagapudi et al., by evaluation of the impact of freeze-dried pomegranate juice (PWE 50–200 μg/ml) on IL-1β-stimulated human neuroblastoma SK-N-SH cells, announced that PWE strongly reduced the production of prostaglandin E2(PGE2), an inhibitor of kappa B (IκB) and IκB kinase (IKK) phosphorylation, and beta-secretase 1 (BACE-1), cyclooxygenase-2 (COX-2), and beta-amyloid (Aβ) expression (Velagapudi et al., 2016).

### Neuro-inflammation

Kim et al., by examination of punicalagin (PL) (1.5 mg/kg) derived from pomegranate in lipopolysaccharide-induced memory impairment in mice, showed that it promoted their function in the water maze, probe, and step-through type passive avoidance tests. PL also alleviated the ranges of Aβ density, amyloid precursor protein (APP), ionized calcium-binding adaptor molecule-1 (Iba-1), glial fibrillary acidic protein (GFAP), and BACE-1 expressions, and IL-6, IL-1β, tumor necrosis factor-alpha (TNF-α), glutathione (GSH), reactive oxygen species (ROS), and malondialdehyde (MDA) profiles in the brain of the mice. Also, PL restrained the activation of nuclear factor kappa B (NF-κB) by restraint of IκB degradation as well as p50 and p65 nuclear translocation (Kim et al., 2017). Based on Stojanovic et al. (2017) study, the hydroethanolic extract of pomegranate peel is full of PU, PL, and ellagic acid (EA), which are synergistically applied together. Jain et al., by investigation of the impact of the extract of Pomegranate on tibial and sural nerve transection (TST)-induced neuropathic pain in rats, demonstrated that PFE improved the mechanical hyperalgesia, cold hyperalgesia, heat hyperalgesia, mechanical dynamic allodynia, and cold allodynia in the rats. Pretreatment with BADGE (a peroxisome proliferator-activated receptor poly (ADP-ribose) polymerase (PPAR)-γ antagonist) and L-arginine (a nitric oxide precursor) antagonized the positive outcomes of PFE. On the other hand, its mixture with L-NAME (a NOS inhibitor) considerably ameliorated the defensive outcomes of PFE against TST-induced neuropathic pain. In conclusion, PFE improves TST-induced neuropathic pain through NO inhibitory, PPAR-γ agonistic activity, anti-inflammatory, and anti-oxidative potentials (Jain et al., 2013). Jain et al. (2021), explored the *in vivo* impact of *P. granatum L*. fruit rind extract and ellagic acid on vincristine-induced neuropathies and exhibited anti-inflammatory and anti-nociceptive properties for *P. granatum L*. fruit rind extract by suppression of TNF-α and interleukin-6 and modification of the GABAergic system. Pomegranate, as a nutraceutical source, has also shown encouraging outcomes in treatment of central neuroinflammation and neurodegenerative processes. It has positive effects on the treatment process of cardiovascular disease, diabetes, obesity, prostate cancer, and intestinal inflammatory disease (Ahmed et al., 2016; Ríos et al., 2018; Alfei et al., 2019; Parisio et al., 2020).

Rojanathammanee et al. (2013) examined the impact of pomegranate extract on transgenic brain-damaged mice developing high amounts of amyloid plaques following APP overexpression. Pomegranate extract markedly extenuated the TNF-α range and nuclear factor of activated T cell 1 (NFATc1) activation in the spleen and brain besides the p-IκB/ IκB ratio in the brain. Additionally, pomegranate extract notably increased the amount of phosphoNFATc2/NFATc2 in the brain of the transgenic mice. Another study investigating the outcomes of the pomegranate fruit (4% w/w) in a transgenic mouse model of AD demonstrated that the pomegranate fruit extenuated the serum profiles of IL-2, IL-3, IL-4, IL-5, IL-9, and IL-10, and brain profiles of Aβ1-40 and Aβ1-42 besides IL-1β, IL-6, and TNF-α ranges in the cortex and hippocampus of the transgenic mice (Essa et al., 2015). BenSaad et al. (2017) found that *P. granatum* derivates, namely ellagic acid, gallic acid, and punicalagin, have anti-inflammatory activities by suppressing stimulated RAW267.4 macrophages, nitric oxide, prostaglandin E2, and outputs. Braidy et al. (2016) studied the effect of the pomegranate fruit (4% w/w) on APPsw/Tg2576 mice and revealed that it decreased the amounts of IL-1β, IL-10, TNF-α, inducible nitric oxide synthase (iNOS), and insulin-like growth factor 1 (IGF-1) and gene expression, and increased brain-derived neurotrophic factor (BDNF) gene expression, phospho-CREB/CREB, protein kinase B (PKB), and mammalian target of rapamycin (mTOR) protein expression in their brain.

In another study, EA strikingly reduced the MDA, protein carbonylation, NO, IL-1β, and TNF-α levels by augmenting GSH level and glutathione peroxidase function in the brain of rats exposed to sodium arsenate. Furthermore, EA improved the equilibrium, motor coordination, and long-term memory in these rats (Goudarzi et al., 2018). It is possible that EA might modify the arsenic-induced neurotoxicity in rats by its antioxidant and anti-inflammatory potentials. Rizk et al. revealed that EA notably decreased the contents of TNF-a, MDA, caspase-3, and iNOS, and conversely augmented GSH levels and monoamines, namely, norepinephrine, dopamine, and serotonin, in doxorubicin-induced neurotoxicity in rats (Rizk et al., 2017). Jha et al. investigated the outcome of EA in streptozotocin-induced sporadic AD in rats. They showed that EA significantly decreased the profile of oxidative stress, pro-inflammatory markers such as GFAP, C-reactive protein (CRP), and acetylcholinesterase (AchE), and Aβ levels (Jha et al., 2018). An investigation on the effect of EA on cuprizone-induced acute demyelination in C57BL/6 J mice revealed that EA decreased the level of IL-17 and, on the contrary, increased IL-11 mRNA expression level in the brain of the mice. Additionally, a high dosage of EA increased the number of mature oligodendrocytes and inhibited their apoptosis (Sanadgol et al., 2017).

### Glioma

Based on Wang et al.'s (2013) study, PL, a polyphenol extracted from pomegranate, obviously decreased the viability of human U87MG glioma cells and provoked their apoptosis by increasing the ranges of cleavage of PARP and activation of caspase-9 and caspase-3.

### Cognitive deficits

In the study conducted by Liu et al., there were 150 male members (74 overweight and 76 with normal weight) aged between 45 and 55 who took either 50 mg EA or placebo cellulose for 3 months. EA reduced the amount of triglyceride, total cholesterol, and low-density lipoprotein (LDL) and increased the range of high-density lipoprotein (HDL) during the 3 months. Although EA elevated the plasma profile of brain-derived neurotrophic factor (BDNF) in the overweight members, it had no influence on the normal-weight group. Additionally, EA elevated the cognition function of the overweight group in the Wechsler Adult Intelligence Scale-Revised and the Montreal Cognitive Assessment. In conclusion, EA improves mild age-related cognitive function (Liu Y. et al., 2018). The flavonoid content of pomegranate is a factor that may contribute primarily to lower hsCRP levels and is suggest to have certain benefits by regulation of the vascular wall including improved endothelial function (Chun et al., 2008; Serafini et al., 2010). A considerable effect of pomegranate supplementation on hsCRP, IL6, and TNFα was also determined. The effect of pomegranate supplementation on CRP, eselectin, MDA, VCAM, and ICAM was not significant (9). In conclusion, pomegranate anti-inflammatory effects on neural cells are arisen by attenuating IL-1β, IL-2, IL-4, IL-5, IL-6, IL-17, COX-2, NO, PGE-2, IFNγ, NF-κB, iNOS, and TNF-α, and enhancing IL-10 and PPAR-γ factors. Pomegranate and EA resist oxidative stress by reducing NO, ROS, and MDA and increasing GSH, SOD, and CAT factors. Also, pomegranate is a useful anti-cancer fruit, which inhibits cell proliferation by decreasing PKB, Bcl-2, mTOR, NF-κB, and COX-2 factors; besides, it provokes apoptosis by increasing Bax, caspase 3, caspase 9, and PARP cleavage.

## Neurotoxicity

Neurotoxicity mediated by acrylamide has been reported in human and animal models (LoPachin, 2004; Parzefall, 2008). The apoptosis and oxidative stress pathways are the main mechanisms of neurotoxicity mediated by acrylamide (Matés et al., 2008; Chen et al., 2013; Park et al., 2019). Foroutanfar et al. (2020) evaluated the protective properties of punicalagin on acrylamide-induced toxicity in rats. Punicalagin improved movement disorders, recovered critical oxidative stress marker levels, and ameliorated apoptosis. Additionally, punicalagin elevated myelin basic protein levels, which were reduced because of acrylamide toxicity. It protects against neurotoxicity mediated by acrylamide *via* antiapoptotic and antioxidant properties. Aluminum is a potent neurotoxic agent that crosses the blood-brain barrier, accumulates in the brain, and contributes to neurodegenerative disorders (Bondy, 2016; Chiroma et al., 2019; Liaquat et al., 2019). Animals exposed to aluminum have shown amyloid-β-protein generation, apoptosis in the brain's hippocampus, and AD symptoms (Kumar et al., 2019). The mentioned disorders may be caused by free radicals and oxidative stress-induced through aluminum exposure (Valko et al., 2007). Abu-Taweel and Al-Mutary (2021) investigated the reduction efficiency of pomegranate juice against aluminum chloride-induced biochemical and neurobehavioral disorders in female mice. Low concentrations of pomegranate juice mediated significant improvements in body weight, spatial learning, memory, oxidative biomarkers, and neurotransmitters in female mice treated with AlCl3. L. Gadouche et al. conducted an investigation on pomegranate juice attenuation effect on neurotoxicity and histopathological changes induced by aluminum in the nervous system of mice. Deposition of Al in the brain reduced cell density that produces states of anxiety, depression, and learning and memory impairments. Treatment with pomegranate juice weakened changes in neural behavior, reduced Al in the brain, and restored histological structure. High-performance liquid chromatography with a diode array detector (HPLCDAD) revealed a variety of bioactive molecules (luteolin, quercetin, and gallic acid) contained in pomegranate. They conclude that eating pomegranate may be neuroprotective for neuropathy caused by Al poisoning (Gadouche et al., 2018).

El-Sayyad et al. investigated the role of atorvastatin and pomegranate juice in improving spinal neurotoxicity in rats, which were maternally fed with a hypercholesterolemic diet. The findings revealed massive cell death and necrosis in the maxillary canal, as well as pyknosis and edematous lesions of nerve cells in the progeny of mothers with hypercholesterolemia. At the hyperfine level, there is evidence of demyelination and vacuolar degeneration of myelinated axons. Many motor and sensory neurons showed condensed chromatin substances in their nuclei. These were related to depletion of the tested neurotransmitters (DA and 5HT) and overexpression of vascular endothelial growth factor, caspases 3 and 7, and 8-hydroxydeoxyguanosine. Elongated DNA damage with overt head and tail regions was detected in neurons from the offspring of mothers with hypercholesterolemia, reducing post-pomegranate replenishment. The pomegranate and atorvastatin supplements showed a clear improvement compared to individual formulations containing atorvastatin or pomegranate, and they were very synergistic (El-Sayyad et al., 2017). Toxicity of the nervous system is accountable for detrimental health effects on the nervous system because of many environmental toxic elements, industrial chemical products, natural toxins, and pharmaceutical medications (Costa et al., 2017). According to research, the ordinary process of healthy aging is connected with cellular and molecular changes in neurons, making them sensitive to metal ion dyshomeostasis, environmental neurotoxicants, deterioration, and disease-specific genetic stressors (Mattson and Magnus, 2006). Gadouche et al. tested the neuroprotective efficacy of pomegranate peel methanolic extract (500 mg kg-1) on lead acetate (1,000 ppm)-caused neurotoxicity in 21 healthy mice. They discovered that co-administration of pomegranate extract with lead reduced locomotion, anxiousness, and depression among lead-exposed mice, as measured by the number of cells traversed, the time spent in the dark section, and the time spent immobile in forced swimming. Pomegranate extract also promoted weight loss and histological changes in the cortex, cerebrum, and hippocampus by lowering lead concentrations in these areas (Gadouche et al., 2020).

## Neuroprotective and vasculoprotective effects

When we age, some changes start to take place in our bodies. These changes begin with subclinical levels and can lead to different neurological diseases (Kowalska et al., 2017). Also, the risk of any vascular disorder increases when we age (Savji et al., 2013). Therefore, it is important to examine the aging process and agents like pomegranate with neuroprotective and vasculoprotective effects. Activation of redox-sensitive genes (ELK-1 and p-JUN) is decreased by pomegranate juice concentrate, and expression of endothelial nitric oxide synthase (eNOS) is raised in cultured human coronary artery endothelial cells that have been exposed to high shear stress *in vitro* (de Nigris et al., 2005). Some defensive functions of the juice of pomegranate were noticed against hydrogen peroxide-induced toxicity in HepG2 models (which is an *in vitro* model system for studies on polarized human hepatocytes) and *Artemia salina* (which is a species of brine shrimp). It also had antiproliferative functions in HeLa and PC-3 cancer cells that inhibit cyclooxygenase-2 (COX-2) and monoamine oxidase (MAO) enzymes (Les et al., 2015). In another report, anti-neuroinflammatory functions of punicalagin (PUN) were noticed by examining microglial BV-2 cells and lipopolysaccharide (LPS)-treated cultured astrocytes. As it was discovered, punicalagin could inhibit impairment of LPS-induced memory through anti-amylogenic and anti-inflammatory methods by inhibiting the nuclear factor kappa-light-chain-enhancer of activated B cells and NF-κB activation (Kim et al., 2017). Clinical studies have illustrated a decrease in diastolic and systolic blood pressures in obese and/or hypertensive patients that received the juice of pomegranate (Lynn et al., 2012; Asgary et al., 2013, 2017; Allahverdian et al., 2014; Haghighian et al., 2016; Moazzen and Alizadeh, 2017). A meaningful decrease in by-products of fat peroxidation and inflammatory biomarkers also occurred with pomegranate juice intake. Pomegranate-containing nutritious supplements were given to a group of patients, and they decreased the levels of diastolic and systolic blood pressures. However, the recovery of cardiovascular risk did not happen (Wu et al., 2015). Pomegranate juice has had positive effects on preservation of the central nervous system (CNS) in some human clinical trials. In pregnant women with intrauterine growth restriction (IUGR), maternal pomegranate juice absorption displayed differences in the neonate brain and structure (Matthews et al., 2019). Other studies suggested that pomegranate juice may be safe as a known IUGR intrauterine neuroprotective agent during pregnancy (Ross et al., 2021). When diabetic rats were given pomegranate juice, reduced inflammatory biomarkers, blood sugar, and lipid levels were noticed (Taheri Rouhi et al., 2017).

A decrease in systemic oxidative stress occurred in pigs that had hypercholesterolemia when pomegranate extract was given to them (Asgary et al., 2017). Supplementation with pomegranate also shows cardiovascular protection. It helps to improve cardiac hypertrophy in cigarette smoke in sight animals (Wang et al., 2018). Improvement in neuronal durability and protection against oxidative destruction in a rat model of Parkinson induced by rotenone were achieved by pomegranate juice treatment (Kujawska et al., 2019). In addition, in a rat model of maternal inflammation, suppression of neuronal nitric oxide synthase and neonatal brain apoptosis, and nuclear factor-κB activation were achieved using pomegranate juice (Ginsberg et al., 2018). The effects of the ingredients of the pomegranate fruit, for instance, ellagic acid, are also concentrated on its protective function in different NDDs. In a review analysis, ellagic acid has been considered as a multi-target pharmacological medicine for the central nervous system (Alfei et al., 2019). Pomegranate metabolites like urolithins suppressed β-amyloid fibrillation *in vitro*, especially methyl-urolithin B (3-methoxy-6H-dibenzo [b, d] pyran-6-one), and had a protective function in *Caenorhabditis elegans* post-induction of amyloid β (1–42)-induced paralysis and neurotoxicity (Yuan et al., 2016). Urolithin A or UA diminished reoxygenation/hypoxia abuse in myocardial cells and reduced myocardial cell death in mice after reperfusion/ischemia. UA led to an increase in antioxidant quantity in cardiomyocytes following reoxygenation/hypoxia that decreased myocardial apoptosis (Tang et al., 2017).

Pretreatment of human umbilical vein endothelial cells (HUVECs) was performed with ellagic acid. Then, incubation with oxidized low-density lipoprotein (oxLDL) was performed. The outcomes showed inhibition of nicotinamide adenine dinucleotide phosphate (NADPH) oxidase, increase in cellular antioxidant defense, and weakening oxLDL-induced Lectin-like oxidized low-density lipoprotein receptor-1 (LOX-1) upregulation and endothelial nitric oxide synthase (eNOS) down-regulation. Lectin-like oxidized LDL (oxLDL) receptor-1 (LOX-1, also known as OLR-1), is a class E scavenger receptor. It intercedes oxLDL uptake through vascular cells. It seems that LOX-1 has an attractive therapeutic purpose for treatment of human atherosclerotic illnesses (Lee et al., 2010). Ellagic acid and punicalagin were used to pretreat adipocyte cells, and that led to inhibition of lipolysis, decreasing MAO function (Les et al., 2017). These studies represented that all parts of pomegranate, along with its chief ingredients such as urolithins, hydrolyzable tannins, and ellagic acid, had a positive effect on lipid levels, blood sugar, neuro/inflammatory biomarkers, and oxidation stress (Trapali and Lagouri, 2021). Hypoxic-ischemic brain injury in newborns is an important cause of perinatal mortality and permanent disability. Estimated incidence is about 2 per 1,000 births (Hankins and Speer, 2003; Shevell, 2004). Loren et al. conducted a study through an animal model of neonatal hypoxic–ischemic brain injury on the neuroprotective effect of maternal nutrition with pomegranate juice. In their study, the participants had free access to drinking water containing pomegranate juice in one of three doses (diluted 1:80, 1:160, and 1:320) as well as vitamin C water, sugar water, and plain water controls during the last trimester of pregnancy. On the 7th day after delivery, the puppy underwent unilateral carotid artery ligation and was subsequently exposed to 8% oxygen for 45 min. Brain damage was histologically evaluated after 1 week of pomegranate juice supplementation, which significantly reduced brain tissue loss (60%) in all the three brain regions investigated, with the highest dose of pomegranate juice being of paramount importance. Pomegranate juice also reduced the activation of caspase 3 by 64% in the cortex and by 84% in the hippocampus. Ellagic acid and polyphenol components of pomegranate juice were detected in the treated puppy plasma but not in control puppies. These findings indicate that maternal supplementation of pomegranate juice protects the newborn's brain (Loren et al., 2005).

## Parkinson's disease

Age is a known risk factor for Parkinson's disease. The average age of onset of the disease is in the 70s. The decreased dopaminergic activity that occurs during aging is assumed to be related to the development of PD's symptoms in the elderly (Hindle, 2010). Accumulation of α-synuclein, a PD hallmark, during the process of aging, disrupts rain synapses. Cellular senescence and impaired cellular function that happen as an individual gets old also contribute to the pathophysiological process of PD (Rey et al., 2021). Investigators treated rat models of rotenone-induced parkinsonism with pomegranate juice to assess the possible neuroprotective potential of pomegranate in PD. The group treated with pomegranate exhibited postural stability improvement and increased neuron survival. Pomegranate juice reduced oxidative stress, lipid peroxidation, and the expression of α-synuclein. The investigators found fewer degenerated neurons in the substantia nigra of the mice that received pomegranate juice (Kujawska et al., 2019). PD is the second most common age-related neurodegenerative disorder after AD. It has several impacts on health, social, and economic conditions that will continue to enhance the population's longevity. Aging is also the most significant risk factor for developing idiopathic parkinsonism (Reeve et al., 2014). As it seems, memory impairment significantly occurs during PD. Administering different doses of pomegranate seed extract to animals with PD improved their memory (Sarkaki et al., 2013). Rezaee et al. examined the possible impact of pomegranate juice on levodopa-induced dyskinesia in mice with PD. Three days of administering levodopa (that is a precursor to dopamine and is often used as a dopamine replacement agent for the treatment process of Parkinsonism) attenuated Parkinson's symptoms, as was seen in abnormal involuntary movement tests. Decreasing effects of levodopa continued up to nearly 2 weeks. Administration of levodopa along with pomegranate juice for the rest of treatment decreased abnormal involuntary movements' scores compared to those continuing to be treated only with levodopa, nearly in a time-dependent manner (Sarah and Mahsa Hadipour, 2018). There was also another article that demonstrated treatment with pomegranate extract, either alone or in combination with levodopa, diminishes the toxic effects of rotenone in PD rats (Kamel, 2020).

**Figure 3:**
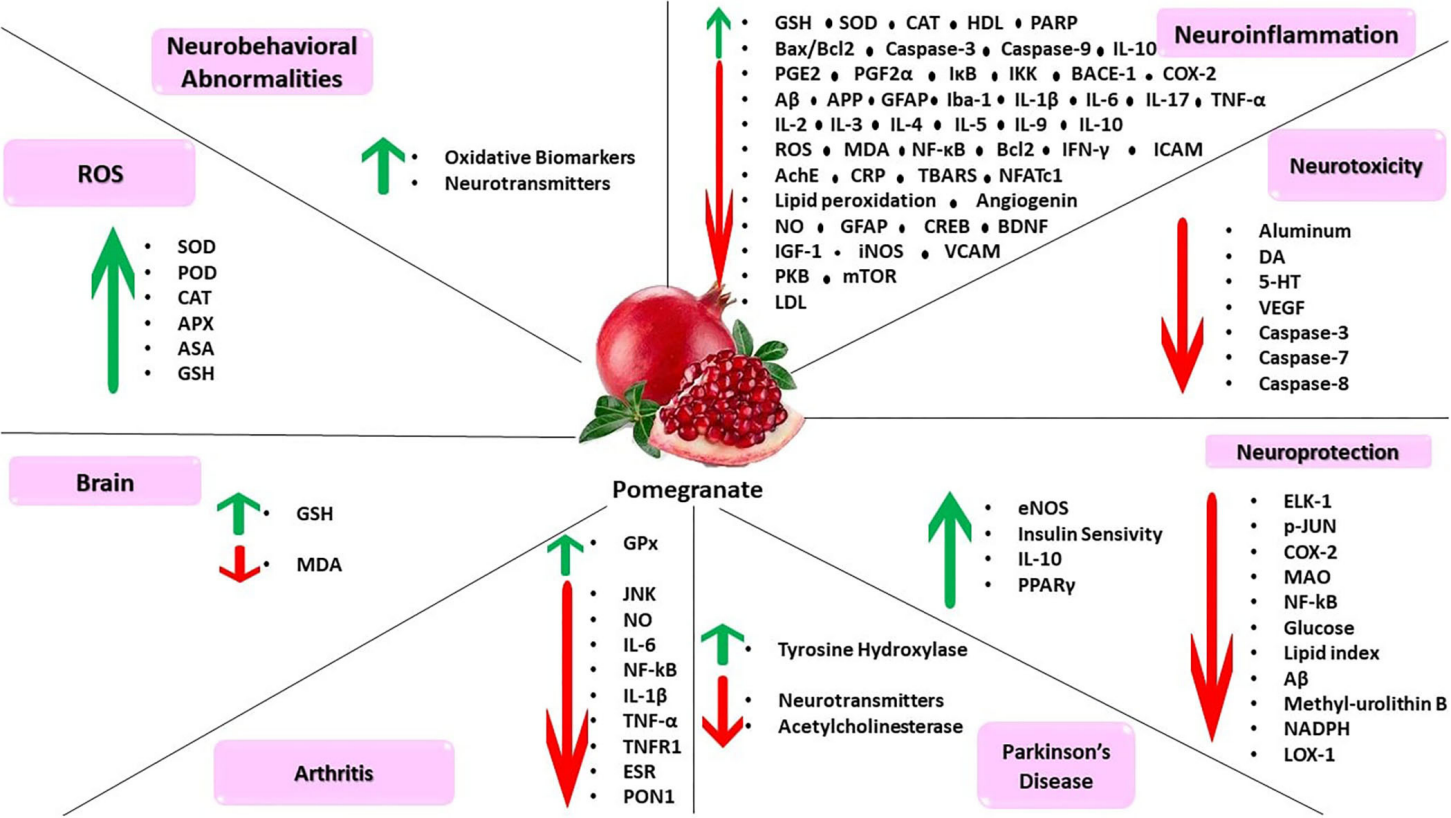
Pomegranate can inhibit neuroinflammation, neurotoxicity, and ROS. This compound has protective agents for brain and age-related neurological disorders such as Parkinson's disease, arthritis, neurobehavioral abnormalities.

In order to examine the lucrative impact of pomegranate juice treatment on survival of neurons, Kujawska et al. performed immunostaining to recognize tyrosine hydroxylase-positive neuron cells in the region of the substantia nigra. The outcome of continuing treatment with rotenone was significant loss of tyrosine hydroxylase-positive neuron cells in comparison with the control group. The treatment process with pomegranate juice alone had no significant effect on the survival of tyrosine hydroxylase cells (Kujawska et al., 2019). Kujawska et al. injected rotenone (that acts as a powerful inhibitor of the mitochondrial complex I) into animals. Consequently, their vertical and horizontal activities were lowered in comparison with the control group. Administration of pomegranate juice to the rotenone-challenged animals diminished the motor deficiency by enhancing vertical activity. Chronic exposure to rotenone reduced dopamine and the level of its main metabolite, 3,4-dihydroxyphenylacetic acid. However, the depletion of midbrain dopamine and 3,4-dihydroxyphenylacetic acid was considerably weakened through mixed treatment with rotenone and pomegranate juice (Kujawska et al., 2021). As an attempt to investigate the plausible proinflammatory function of pomegranate juice, Tapias et al. examined the expression of the nuclear factor-kappa B subunit p65, tumor necrosis factor-α, enzyme cyclooxygenase-2, and cytokine interleukin-1β in the substantia nigra of rats exposed to rotenone. As was displayed by Western blots, pomegranate juice administration did not modify cyclooxygenase-2 expression, and levels of Interleukin-1β protein were also unchanged as a response to the combination of pomegranate juice and rotenone. However, the administration of pomegranate juice led to a massive growth in the p65 catalytic subunit of the transcription nuclear factor kappa B following rotenone injury (Tapias et al., 2014). Fathy et al. investigated the protective effect of pomegranate juice and pomegranate seed extract against paraquat-induced neurotoxicity in mice (Paraquat is a herbicide that can cause Parkinsonian-like signs in animals and men). The protein level of tyrosine hydroxylase was evaluated in the substantia nigra in order to examine the dopaminergic neuronal loss in different experimental groups. The level of tyrosine hydroxylase was considerably decreased in the region of the substantia nigra of the Paraquat (alone)-induced group in comparison with the control group. Oral treatment with either pomegranate juice or pomegranate seed extract resulted in significant growth in the protein expression of tyrosine hydroxylase than the Paraquat (alone)-treated mice (Fathy et al., 2021). For the purpose of examining the plausible impact of pomegranate in combination with propolis, vinpocetine, or cocoa in the treatment process of PD even without being given as an adjuvant to levodopa, Azza et al. performed a number of behavioral analyses on a rat model of parkinsonism. They found that treating with levodopa alone or with all the pomegranate combinations decreased the deficiencies in inflammatory markers, locomotor activities, cognition, levels of neurotransmitters, acetylcholinesterase activity, and oxidative stress (Ali et al., 2022) (Figure 3).

## Ischemia

Age is among the unmodifiable risk factors for stroke. More than 70% of strokes happen in people older than 65, and the incidence rate of the disease significantly increases after the age of 45 (Edzie et al., 2021). The disease also has a poorer prognosis in older patients than in the young. It is assumed that ANDs and changes in the CNS are related to poor prognosis of stroke in the elderly (Chen et al., 2010). The presence of comorbidities is more common in older adults. Thus, aiming at controlling comorbid conditions is beneficial for increasing the survival rate (Michael and Shaughnessy, 2006). Pre-administering pomegranate extract in rat models of ischemia/reperfusion (I/R) showed a reduction in TNF-α, nitric oxide (NO), malondialdehyde (MDA), NF-κB (nuclear factor kappa B p65), and caspase-3 levels in the rats' brain. Furthermore, it also enhanced the activities of antioxidant enzymes GRD, GPX, and SOD. Brain injury assessment by comet assay showed that mice that consumed pomegranate had less DNA damage in their brains. Due to the protective effect of pomegranate extract on the brain, the researchers suggested pomegranate as a beneficial substance in patients at risk of stroke (Ahmed et al., 2014b).

Ahmed et al. conducted an investigation, Pomegranate Extract Protection Against Cerebral Ischemia/Reperfusion Injury and Preserves Brain DNA Integrity in Rats. This study was conducted to investigate the protective effect of standardized pomegranate extract on cerebral ischemia/reperfusion-induced brain injury in rats, with standardized pomegranate extract for the control group and ischemia/reperfusion (I / R) group at two doses (250 and 500 mg / kg) administered for 15 days prior to ischemia-reperfusion. After I/R or sham surgery, all the rats were sacrificed, and their brains were harvested for subsequent biochemical analysis. The results showed decreased brain levels of MDA and NO in addition to enhanced SOD, GPX, and GRD activity in the rats treated with pomegranate extract prior to the brain I/R. In addition, pomegranate extract reduced the brain levels of NFκB p65, TNFα, and caspase 3 and increased the brain levels of IL10 and brain ATP production. The comet assay showed less brain DNA damage in the rats protected with pomegranate extract. This study was the first to show this pretreatment. The amount of pomegranate extract in rats may provide significant dose-dependence antioxidant, anti-inflammatory, anti-apoptotic, and neuroprotective effects on the brain I/R brain injury and DNA injury because of ATP supplementation (Ahmed et al., 2014b). West et al. conducted an investigation, Pomegranate Polyphenols and Resveratrol Protection of the Neonatal Brain against Hypoxic-Ischemic Injury. One of the best-studied polyphenols is resveratrol, which is found in grapes, some nuts, and kojohkon (Japanese hollyhock) and an oriental plant drug used to treat vascular diseases (Sato et al., 1997; Faustino et al., 2003; Tokuşoglu et al., 2005). Resveratrol is an antioxidant, but its effects on the body far outweigh those of other antioxidants. West et al. discovered that pomegranate polyphenol extract and resveratrol can protect the neonatal rodent brain from HI brain injury. Resveratrol, when administered prior to the injury, can protect against both caspase-3 activation 24 h after the injury and tissue loss 7 days after the injury. They suggest that polyphenols need to be considered for further evaluation as potential treatment to reduce the effects of neonatal HI brain injury (West et al., 2007).

## Brain

Belal et al. studied the possible protective effect of pomegranate peel extract on structural changes in rat brains, which are mediated by mobile phone radiation. Thirty adult male rats were divided into five groups. Group I was the control, group II rats were exposed to 900 MHz, and group III rats were exposed to 1,800 MHz for 2 months. Group IV rats were exposed to 900 MHz and pomegranate peel extract (500 mg/kg) at the same time, and group V rats were orally administered 1,800 MHz and pomegranate peel extract (500 mg/kg) in an aqueous solution once daily. The frontal cortex and cerebellar tissues were resected and treated for histopathological and immunohistochemical studies. The cerebral and cerebellar cortices of the rats exposed to mobile phone radiation showed degenerative changes, especially in neurons. This study concluded that pomegranate extract can improve histopathological changes induced by cellular electromagnetic radiation (Belal et al., 2020). Kandeil et al. conducted research on the protective effect of pomegranate peel extract on titanium dioxide nanoparticles. Forty male albino rats were divided into 4 groups: the control group; the TiO2NP group: rats that were orally administered TiO2NPs on days 17–30; received pomegranate peel extract group: rats that received pomegranate peel extract for 30 days; rats in the pomegranate peel extract + TiO2NPs group received the same dose of TiO2NPs for 1 h, and were previously administered pomegranate peel extract. The potent antioxidant activity of pomegranate peel extract was demonstrated by both Nrf2 and NQO1 mRNA expressions, significant increase in glutathione concentration, inducible nitrogen monoxide synthase mRNA expression, and significant decrease in malondialdehyde concentration. They concluded that dopamine, serotonin levels, acetylcholinesterase, and catalase activity returned to normal. These findings were confirmed by histopathological data showing severe degeneration, edema, and congestion that spread to the brain tissue of TiO2NP, and these features were ameliorated by pomegranate peel extract administration. Pomegranate peel extract is a potent antioxidant that can effectively increase the transcription of Nrf2 and NQO1 in TiO2NP-induced brain toxicity (Kandeil et al., 2018).

The creation of cognitive and behavioral functions requires the complicated but essential process of human brain development (Stiles and Jernigan, 2010). The prenatal period is the source of many neurodevelopmental abnormalities in motor and cognitive functions (Smith, 2000). Matthews et al. studied the effect of maternal pomegranate juice administration on IUGR pregnancies with the structure and function of a newborn's brain. Their study found no differences in brain damage, measurements, or volumes between groups, but they managed to find that the treatment subjects had lower diffusivity in the anterior and posterior limbs of the internal capsule compared to the controls, and that resting-state functional connectivity displayed greater correlation and covariance in multiple networks in treatment cases, with alterations most visible in the visual network in per-protocol analyses (Matthews et al., 2019). Traumatic brain injury (TBI) is frequently induced by stressful conditions, which can result in symptoms of post-traumatic stress disorder (PTSD) and neurobehavioral symptoms of brain damage (Klyce et al., 2021). Daradkeh et al. investigated the impact of pomegranate juice intake on nutritional and behavioral results following traumatic brain damage. Those with poor behavioral scores progressed to the treatment process. i.e., supplemented with 250 ml/day of pomegranate juice and monitored every 3 months for a year. They discovered that drinking pomegranate juice might enhance behavioral results (Daradkeh, 2017). When someone has difficulty remembering, learning new things, focusing, or making judgments, this is referred to as cognitive impairment (Kobayashi et al., 2021). Although cognitive impairment without functional restrictions has numerous causes, it frequently leads to dementia or Alzheimer's disease in elderly adults, especially if memory is affected (Plassman et al., 2011). McIlorum et al. conducted research to see if 250 ml of Biona pomegranate juice enhances executive cognitive performance in men aged 18 to 35 who are in danger of mild brain trauma. They demonstrated that acute supplementation with both Biona pomegranate juice and Volvic berry medley juice reduced reaction times, although the findings were not significantly different (McIlorum, 2018).

## Reactive oxygen species

Reactive oxygen species (ROS) are free radicals that include the hydroxyl radical (•OH) and the superoxide anion (O2•-). Hydrogen peroxide (H2O2) is another ROS; however, it is a non-radical molecule that is abundant and more reactive in higher plants (Gill and Tuteja, 2010). ROS perform a dual role in plants, both useful and harmful depending on their concentration. ROS have beneficial impacts such as acting as a signaling molecule at low levels and causing adverse effects such as cell death at high levels (Khalvati et al., 2010; Miller et al., 2010). Because of balance between ROS generation and removal in plants, ROS operate as signaling molecules at low levels, controlling numerous physiological and developmental activities, and as destructive chemicals at high concentrations (Mittler et al., 2004).

## ROS scavenging process by pomegranate

Plants have innate defensive mechanisms that allow them to withstand stress. These include non-enzymatic and enzymatic antioxidant defense systems. When ROS are created in a steady state, the antioxidant defense system prevents them from causing damage (Foyer and Noctor, 2005; Navrot et al., 2007). The enzymatic antioxidant defense system consists of superoxide dismutase (SOD), peroxidase (POD), catalase (CAT), and ascorbate peroxidase (APX). The non-enzymatic antioxidant system consists of non-enzymatic low molecular metabolites such as phenolic compounds, carotenoids, tocopherol, ascorbate (ASA), and glutathione (GSH) (Chaves and Oliveira, 2004; Mittler et al., 2004; Becana et al., 2010).

### CAT

This enzyme is also known as H2O2 oxidoreductase (Karuppanapandian et al., 2011). According to one study, there was a reduction in CAT activity with pomegranate in the presence of high salinity, and a decrease in CAT activity showed that CAT offered resistance against salt stress with minimal protection. The trend in SOD activity in pomegranate leaves was initially raised and then reduced. One research revealed that CAT activity was increased in several pomegranate species when exposed to water stress, with the exception of one (Pourghayoumi et al., 2017; Liu C. et al., 2018).

### SOD

SOD is primarily responsible for oxidative stress defense in all aerobic species (Scandalios, 1993). It has been found that when plants are subjected to different environmental conditions such as drought and metal toxicity, SOD activity rises (Sharma and Dubey, 2005; Mishra et al., 2011). It has been observed that increased SOD production improves plant tolerance to oxidative stress (Gupta et al., 1993). Plants are more tolerant of environmental stressors when SOD activity is enhanced (Sharma et al., 2012). According to one study, there was a reduction in SOD activity with pomegranate in the presence of high salinity, and the decrease in SOD activity showed that SOD offered defense against salt stress with limited protection. The trend in SOD activity in pomegranate leaves was initially raised and then reduced (Liu C. et al., 2018). One research revealed that SOD activity rose dramatically in multiple pomegranate species during water stress, with the exception of one of them (Pourghayoumi et al., 2017). Mastrogiovanni et al. (2021), in a study on bovine peripheral blood mononuclear cells, demonstrated that pomegranate peel extract had a potent anti-proliferative effect induced by concanavalin A and an anti-oxidative moderate effect induced by hydrogen peroxide or a lipopolysaccharide. Cisplatin is a well-known chemotherapeutic medication that can be used as a combined therapy in brain tumors, but it has nephrotoxic effects inducing oxidative stress and inflammation. Harakeh et al. investigated the impact of PE-NPs (pomegranate enclosed by nanoparticle to improve its solubility and bioavailability) on cisplatin-associated nephrotoxicity in a mice model of Ehrlich solid carcinoma (ESC). They demonstrated that PE-NPs notably increased antioxidants such as SOD, GSH, and CAT. It also reduced the ranges of NF-kB, IL-1β, and TNF-α, which are involved in cisplatin-induced inflammation. Therefore, PE-NPs can be a potent cisplatin adjuvant therapy (Harakeh et al., 2022).

According to one study, the antioxidant functions of pomegranate peel are greater than in other parts, so it may be helpful for human health in therapeutic uses (due to the medicinal properties of its substances) and commercial products such as food storage for a longer duration. However, antioxidants in various areas of the same plant may react differently to multiple conditions. There is still no study performed to evaluate the level of non-enzymatic antioxidants under different stressful situations (Aslam, 2022).

## Neurobehavioral abnormalities

The ability to maintain stability and adaptability in the face of task limits is referred to as behavioral adaptation. These two apparently contradictory qualities may be influenced by age-related changes in the system component parts and their interactions (Vaillancourt and Newell, 2002). Abu-Taweel et al. investigated the efficacy of pomegranate juice in alleviating aluminum chloride-induced biochemical and neurobehavioral abnormalities in female mice. In AlCl3-treated female mice, pomegranate juice, particularly at low dilutions, improved spatial memory, weight, and learning throughout shuttle box tasks, T-maze, and Morris water maze, as well as oxidative biomarkers and neurotransmitters (Abu-Taweel and Al-Mutary, 2021).

## Conclusion

Aging's molecular process involves genome-wide changes like genomic instability caused by mutation accumulation, telomere attrition, and epigenetic modifications. These changes accumulate throughout the course of an organism's existence, eventually resulting in morphological and functional degradation. The brain appears to be particularly susceptible, as neurons do not divide and their reserve shrinks with time. The senescent brain's structural alterations primarily impact the cerebral WM and GM; these consequences include gradual neuronal loss, decreasing levels of neurotransmitters, increased inflammatory responses, and impaired integrity of arteries and the BBB, which leads to infarction and microbleeds. These alterations may result in degenerative disorders such as Parkinson's disease and dementia. Malnutrition and malabsorption syndrome, which are common in the elderly, may result in lower quantities of vitamins required for Hcy metabolism. As a result, the cerebral vasculature sustains more damage, resulting in degeneration and strokes. Thereby, gradual age-related vascular damage to the brain occurs, which is also linked to increased occurrence of epilepsy in the elderly. A rise in the incidence of brain tumors may be noticed in old age, most likely as a result of the decreased efficacy of repair processes. It has been demonstrated that inactivation of genes essential in DNA repair progresses with age. Such changes, whether caused by epigenetic modifications or mutations, may further destabilize immunologic systems and cellular repair processes, increasing vulnerability to ROS and spontaneous mutation and leading to uncontrollable cellular proliferation and age-related neoplasia.

A great deal of studies has been conducted on the therapeutic potential of several compounds for prevention of age-related diseases; in this regard, pomegranate is one of the most actively studied ones. Pomegranate has been shown in animal and clinical tests to be effective in treatment of Alzheimer's disease by reducing amyloid-beta and decreasing atherosclerotic plaque development by decreasing serum lipid levels, and it may help in preventing NDDs caused by oxidative stress and inhibiting aluminum-induced oxidative stress. Many studies have found that pomegranate can reduce the levels of IL-6, IL-1, tumor necrosis factor-alpha (TNF-), reactive oxygen species (ROS), glutathione (GSH), and malondialdehyde (MDA), making it anti-inflammatory and anti-oxidative. Other research has revealed that pomegranate may decrease the viability of human U87MG glioma cells and improve mild age-related cognitive function, increase memory in aged individuals, enhance behavioral results, show cognitive improvement, and has positive activities of daily living in MS patients. It has also been demonstrated that pomegranate has significant dose-dependent antioxidant, anti-inflammatory, anti-apoptotic, neuroprotective effects on brain and DNA injuries, significant dose-dependent anticonvulsive activity, and ability to neuroprotect the newborn's brain and reduce the effects of neonatal HI brain injury. So far, the results have been very promising, so we suggest that pomegranate could be used as a beneficial supplemental therapy. Additional RCT research on the effect of pomegranate compounds on human diseases, as well as more *in vitro* and *in vivo* investigations, should be conducted to uncover more underlying mechanisms of pomegranate compounds in various diseases.

## Author contributions

Study concept, design, critical revision of the manuscript for important intellectual content, and study supervision: ND. Acquisition of data: ME, SAs, NT, PD, HA, and SAk. Drafting of the manuscript: ME, SAs, NT, PD, HA, SAk, ZS, MM, DA, FA, MS, GE, MN, MK, and SH. Revision: SH, MN, and MK. All authors contributed to the article and approved the submitted version.

## Conflict of interest

The authors declare that the research was conducted in the absence of any commercial or financial relationships that could be construed as a potential conflict of interest.

## Publisher's note

All claims expressed in this article are solely those of the authors and do not necessarily represent those of their affiliated organizations, or those of the publisher, the editors and the reviewers. Any product that may be evaluated in this article, or claim that may be made by its manufacturer, is not guaranteed or endorsed by the publisher.
